# The complete mitogenome of the *Chionoecetes opilio* (Crustacea: Decapoda: Oregoniidae) and its unique characteristics

**DOI:** 10.1080/23802359.2020.1780974

**Published:** 2020-06-24

**Authors:** Jin-Hyeop Jeong, Seongho Ryu, Won Kim

**Affiliations:** a School of Biological Sciences, Seoul National University, Seoul, Korea; b Soonchunhyang Institute of Medi-bio Science (SIMS), Soonchunhyang University, Cheonan, Korea

**Keywords:** Mitogenome, Decapoda, Majoidea, *Chionoecetes opilio*, atypical tRNAs

## Abstract

The complete mitochondrial genome of *Chionoecetes opilio* is a 16,067 bp long, circular molecule which contains 13 protein-coding genes (PCGs), 22 transfer RNA genes (tRNAs), and 2 ribosomal RNA genes (rRNAs). Its gene contents and organization are generally similar to other majoid mitogenomes. However, the mitogenome shows unique characteristics; long terminal amino acids, loss or addition of 3 PCGs, a 1216 bp long putative D-loop region, and peculiar secondary structures of 5 tRNAs. The concatenated amino acid sequences of 13 PCGs were used to analyze the phylogenetic tree, which well supported the monophyly of brachyuran clades of Majoidea, Heterotremata, Thoracotremata, and Eubrachyura.

Snow crabs are famous food crab species belonging to the Genus *Chionoecetes* Krøyer, 1838, which inhabit the waters of the Northern Pacific and the Northwestern Atlantic regions (Alvsvåg et al. 2009**;** Ng et al. 2008). Due to its largest annual catches, *Chionoecetes opilio* is the most important commercial species among the congeneric species (FAO Fisheries and Aquaculture Department 2019). Despite of its economic importance, sequenced mitochondrial genomic resource of *C. opilio* is not available yet. Here, we report the first complete mitochondrial genome of the *C. opilio*.

A male individual of *C. opilio* was collected at the offshore of Yeongdeok-gun (the East Sea, South Korea) on 14 March 2019, and then deposited in Marine Arthropod Deposit Bank Korea (MADBK) at the Seoul National University (voucher number MADBK_172402_WGS). The whole genomic DNA was prepared from its muscle using the phenol-chloroform extraction and then sequenced by Illumina HiSeq X Ten. The sequenced reads were filtered, and then 10,000,000 filtered reads were randomly sampled. For *de novo* assembly and annotation, MitoZ software (Meng et al. 2019) and MITOS webserver (Bernt et al. 2013) were applied. The annotated protein-coding genes (PCGs) were manually revised when a significant misalignment was found by blast similarity searching. To further compare the gene organization and content, mitogenomes of 4 Majoidea species were downloaded from NCBI GenBank (https://www.ncbi.nlm.nih.gov/genbank/).

The complete *C. opilio* mitogenome (GenBank accession number, MT335860) consisted of 16,067 bp circular nucleotides and 37mitochondrial genes (13 PCGs, 22 tRNAs, and 2 rRNAs). The GC content for the whole mitogenome is 28.40%, and both AT and GC-skew are negative while GC-skew shows strongly negative value (–0.226) compared to that of AT-skew (–0.032). PCGs generally show negative AT and GC-skew, and 4 NADH dehydrogenase subunit genes (*nd1*, *nd4*, *nd4l*, and *nd5*) located on the (−) strand shows positive GC-skew which are general features of arthropod mitogenomes (Pisani et al. 2013).

Although generally similar to other majoid mitogenomes, *C. opilio* mitogenome has unique characteristics. The mitogenome has 6 overlaps between the genes spanning 1–7 bp. There are unusual losses or additions of long amino acids at 5′ or 3′ ends in products of 3PCGs (ND4, ND4L, and ND1). The 5′ amino acids deletions are found from ND4L (6aa long, 5′ MMDLSF missing). The 3′ additions are found from ND4 (10aa long, 3′ SLIKMKCVKR). The 3′ end replacements are found from ND1 (LNLIFN to WI). There is a putative D-loop region between *rrnS* and *trnI* similar to other majoid mitogenomes; however, its length is longer (1216 bp) when compared to those of other brachyurans (Shi et al. 2015; Márquez et al. 2016; Basso et al. 2017; Karagozlu et al. 2018; Kim et al. 2019).

Generally, *C. opilio* tRNAs show common cloverleaf-shaped secondary structures, and all 22 tRNAs lack variable arms. However, 5 tRNAs have atypical secondary structures; TψC arm without the loop (*trnF* and *trnR*), 1 bp mismatch at the acceptor or anticodon stem (*trnK* and *trnW*, respectively). In addition, the DHU arm of *trnS1* is extremely reduced with short stem (1 bp) and loop (3 bp).

The organizations of mitochondrial genes among majoid mitogenomes are generally conserved, with the almost identical gene synteny starting from *cox1* to *trnE*. While *C. opilio*, *C. japonicus* and *Damithrax spinosissimus* show the almost identical synteny, there is a gene order rearrangement from *Maja crispata* and *Maja squinado* genomes (putative translocation of *nd6*-*cytb*-*trns2* segment between *trnE* and *nd1*).

The complete mitogenomes of 10 Brachyura species (4 Majoids, 2 non-majoid Heterotremes, 2 Thoracotremes, 2 Raninoids), and one outgroup (*Clibanarius infraspinatus*) were downloaded from the GenBank and used to reconstruct their phylogenetic relationships. The concatenated amino acid sequences of 13 PCGs were analyzed by RAxML 8.2.12 (Stamatakis 2006) using the maximum-likelihood (ML)method and MrBayes 3.2.7 (Ronquist et al. 2012) using Bayesian inference (BI) analysis. The consensus tree based on both ML and BI analyses results supports the monophyly of the Majoidea, Heterotremata, Thoracotreamata, Eubrachyura, and Raninoidea with 100% bootstrap values and 1.00 posterior possibilities (Figure 1).

**Figure 1.**
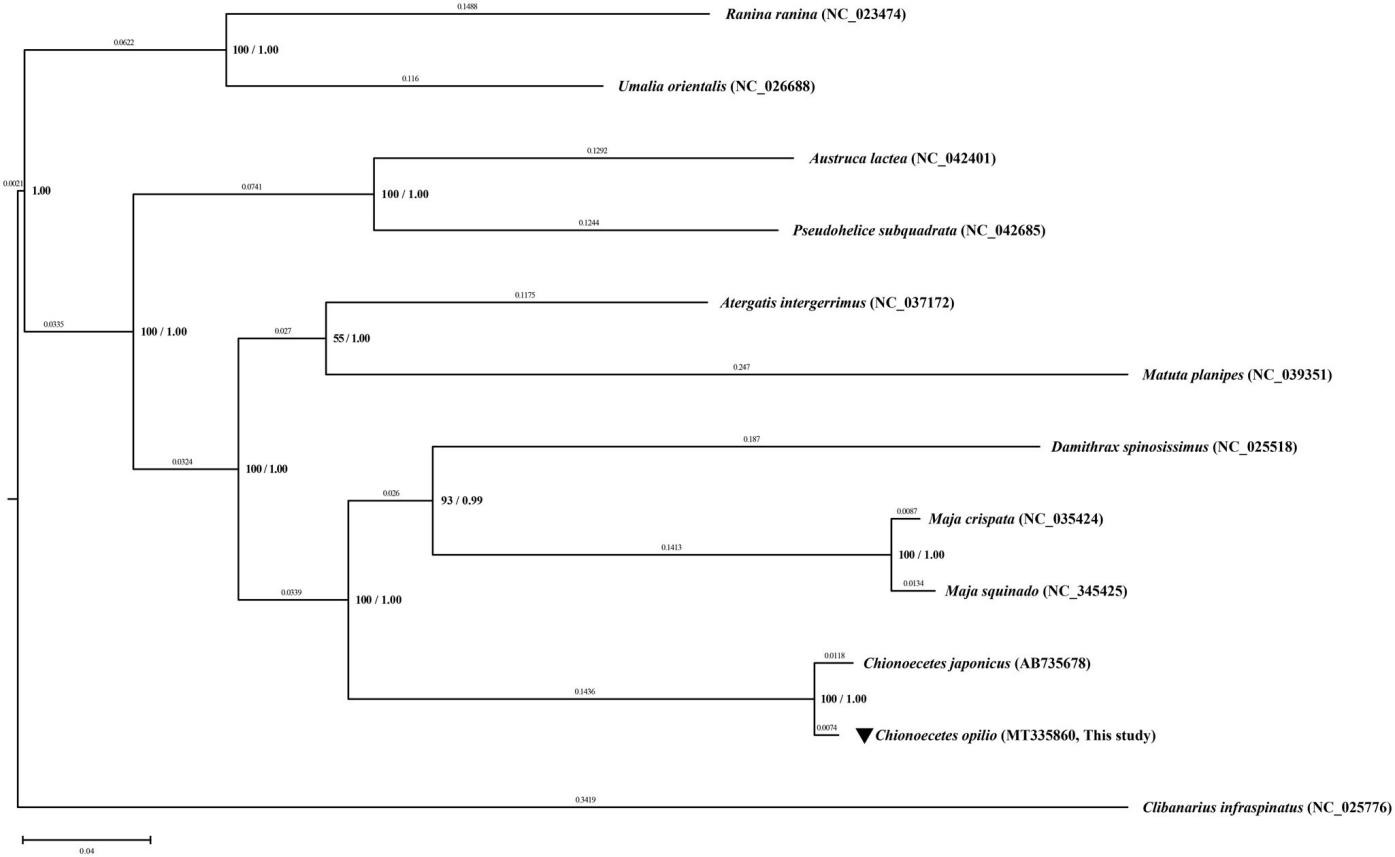
The phylogenetic tree showing relationships between *Chionoecetes opilio* and 11 brachyuran species with an outgroup taxon (*Clibanarius infraspinatus*). The tree was reconstructed from the concatenated amino acid sequences of 13 PCGs using RAxML 8.2.12 and MrBayes 3.2.7 applications based on the ML method. The bootstrap value above 50% in the ML analysis and posterior probability above 0.90 from the BI analysis are indicated at the bases of the each node. The distance based on the BI analysis is indicated above each node. GenBank accession number for each species is indicated with its respective scientific name. The species of interest of this study, *C. opilio* is marked with a reversed triangle.

## Data Availability

The data that support the findings of this study are openly available in GenBank of NCBI at https://www.ncbi.nlm.nih.gov/nuccore/MT335860.
